# Chemistry *(Animal)*

**Published:** 1818-04

**Authors:** 


### II. CHEMISTRY (*Animal*).

*Extraordinary Composition of Human Urine.* The following curious account of a "peculiar urine voided by a Creole of the Isle of France,"* has been lately published by M. Chatelain, in oue of the French Journals. The subject of the observation was aged 40, married, apparently *quite healthy*, and had borne no children for ten years. This urine had the whiteness, consistence, and opacity of milk. It was neither acid nor alkaline; for it in no way changed the litmus or turmeric paper, even after a contact of some hours. It was nearly inodorous, and imparted to the organ of taste a sweetish, perceptibly salt, but not disagreeable savour. Its density, compared to that of distilled water, was 20 : 19; consequently somewhat above that of healthy human urine; the specific gravity of which varies from 100 to 103—that of distilled water being 100.

Left in a temperature of from 14° to 15° (57°—59° Faht.) this animal fluid separated into two distinct parts; a superior, white and opaque; and an inferior semi-transparent, and exhibiting all the physicial characters of whey spontaneously formed. Alcohol at 40°, poured on the urine, produced a white, light, copious precipitate, soluble in ammonia. Treated with an excess of concentrated sulphuric acid, it manifested a slight effervescence without precipitation, and assumed a faint rose-coloured tint. Liquid ammonia effected in it no sensible change. Exposed to the heat of a sand bath, it yielded, in a few moments, a white firm coagulum; which weighed, while moist, $\frac{1}{24}$ of the urine employed. This coagulum, dried between coarse paper, and then placed on live coals, yielded a sort of crepitation, liquified, exhaled, as it burnt, an ammoniacal odour, and left a coal-like residium It was scarcely soluble in distilled and boiling acetic acid; but more readily so in the diluted sulphuric, to which it imparted a rose colour. It formed with potash and ammonia, solutions which constantly retained a milky appearance, and precipitated by the addition of the diluted acids Its solution in water of potash, submitted to distillation, exhibited the most decided traces of ammonia.

When freed by the action of caloric from this animal matter, to which were owing its whiteness, consistence, and opacity, the urine

---

* *Notice sur une urine particulière rendue par une Créole de l' ile-de-France, &c.; par M. Chatelain. Bulletin de la Société Medicale de Emulation.* No. vi. Juin, 1817.

became transparent and nearly colourless. Reduced to the sixth of its primitive volume, it was found, by the ordinary processess, to contain the alkaline muriates and sulphates, phosphates of magnesia, and urea, nearly in the proportions in which these substances exist in the urine of the healthy subject; but the presence of the uncombined uric and phosphoric acids, and of the phosphate, could not be detected; nor that of any saline combination having ammonia for its base.

From the preceding experiment, made on only fifteen ounces of the urine, it results that this animal fluid differed widely from that which is voided in the natural state, principally by the presence of a peculiar azoted substance, possessing apparently a great analogy with cheese or cheesy matter; by the absence of all uncombined acid; and lastly, by the non-existence of the calcareous phosphate and ammoniacal salts.

M. Chatelain leaves it to physiologists to explain the formation of this animal matter, coagulable by heat, and soluble in ammonia, potash, and the diluted mineral, and concentrated vegetable acids; and which may be the same as that found by Caballe, in the milky urine, of which he has published the analysis.* Yet the urine which this chemist subjected to experiment, and which had been voided by a young widow, contained, in addition to the animal substance above mentioned, all the materials composing healthy human urine; wherein it differs essentially from that which constitutes the object of this notice.

